# A parameter estimation algorithm for LFM/BPSK hybrid modulated signal intercepted by Nyquist folding receiver

**DOI:** 10.1186/s13634-016-0387-2

**Published:** 2016-08-18

**Authors:** Zhaoyang Qiu, Pei Wang, Jun Zhu, Bin Tang

**Affiliations:** School of Electronic Engineering, University of Electronic Science and Technology of China, Chengdu, China

**Keywords:** Nyquist folding receiver, LFM/BPSK hybrid modulated signal, Parameter estimation, Signal characteristics

## Abstract

Nyquist folding receiver (NYFR) is a novel ultra-wideband receiver architecture which can realize wideband receiving with a small amount of equipment. Linear frequency modulated/binary phase shift keying (LFM/BPSK) hybrid modulated signal is a novel kind of low probability interception signal with wide bandwidth. The NYFR is an effective architecture to intercept the LFM/BPSK signal and the LFM/BPSK signal intercepted by the NYFR will add the local oscillator modulation. A parameter estimation algorithm for the NYFR output signal is proposed. According to the NYFR prior information, the chirp singular value ratio spectrum is proposed to estimate the chirp rate. Then, based on the output self-characteristic, matching component function is designed to estimate Nyquist zone (NZ) index. Finally, matching code and subspace method are employed to estimate the phase change points and code length. Compared with the existing methods, the proposed algorithm has a better performance. It also has no need to construct a multi-channel structure, which means the computational complexity for the NZ index estimation is small. The simulation results demonstrate the efficacy of the proposed algorithm.

## Introduction

Currently, the electromagnetic environment is becoming increasingly complex and many modern radar signals have very high carrier frequencies or wide operating bandwidths [1, 2]. In order to intercept the modern radar signals, some receiver architectures have been proposed in the past few decades [3, 4]. The wideband non-cooperative receivers should have the capability of wideband receiving. A typical wideband receiver is the channelization structure, which adopts a set of analog band-pass filters to reduce the bandwidth of each channel and samples each channel with a low-speed analog to digital converter (ADC) using filter bank [4]. However, this kind of structure needs a huge amount of equipment. For the purpose of realizing wideband monitoring with a small amount of equipment, the Nyquist folding receiver (NYFR) architecture is proposed and it can realize wideband monitoring using one ADC [5, 6]. The NYFR modulates the received analog signal in the front-end of the receiver, maps the Nyquist zone (NZ) information to the modulation bandwidth of the signal, and then samples the modulated signal.

Based on the NYFR structure, the output signal processing using wavelet transform has been studied [7]. Then, some new NYFR architectures using different local oscillator (LOS) modulation types have been proposed. Synchronous NYFR (SNYFR) structure using simplified LOS has been proposed and its output can be processed more easily because of the synchronous LOS [8]. Other LOS modulation types such as binary phase shift keying (BPSK) LOS and noise sequences are proposed [9, 10], which can improve the performance of NYFR because the bandwidths of these LOS modulations remain unchanged.

The NYFR can realize wideband receiving with a small amount of equipment, but the information of LOS modulation will be added on its output [8], and its output will be more complex compared with the conventional receiver. Some conventional radar signals such as linear frequency modulation (LFM) signal and frequency agile (FA) signal intercepted by the NYFR have been investigated, and the parameter estimation methods using multi-channel structure have been proposed [8, 11].

Meanwhile, many low probability interception (LPI) radar waveforms have been designed. Linear frequency modulated/binary phase shift keying (LFM/BPSK) hybrid modulated signal is a novel kind of LPI radar signal. It has a double spread spectrum and has been applied in some radar and fuse systems [2]. For the parameter estimation of LFM/BPSK signal intercepted by the conventional receiver, an algorithm based on Zhao, Atlas, and Marks (ZAM) transformation has been studied [12]. However, for the parameter estimation of LFM/BPSK signal intercepted by the NYFR, there has been no public report.

Therefore, considering the increasing complexity of radar waveform and the growing demand of wideband receiving, it is necessary to study the parameter estimation of LFM/BPSK signal intercepted by the NYFR. The LFM/BPSK signal intercepted by the NYFR is a typical non-stationary signal. For a non-stationary signal, a common processing idea is the time-frequency analysis. However, many time-frequency methods can achieve optimal results only for the particular modulation types [13]. Because the LFM/BPSK signal intercepted by the NYFR contains the LOS modulation, it may be difficult to find a time-frequency kernel which is optimal for the NYFR output directly. In this paper, we will study this problem in another way and make full use of the NYFR prior information which is neglected in [8] and [11]. We will model the LFM/BPSK signal intercepted by the NYFR based on the signal self-characteristic and the NYFR prior information, and propose a parameter estimation algorithm which has different estimation steps compared with the existing NYFR output parameter estimation algorithm [8, 11].

This paper is organized as follows: Section 2 investigates the NYFR architecture and the LFM/BPSK hybrid modulated signal intercepted by the NYFR. Section 3 gives the parameter estimation methods for each modulations of the NYFR output. Section 4 is the algorithm steps for the parameter estimation of the NYFR output. Section 5 gives the simulation results and the corresponding analyses, and we conclude in Section 6.

## NYFR architecture and NYFR output signal analysis

### NYFR architecture

**Fig. 1:**
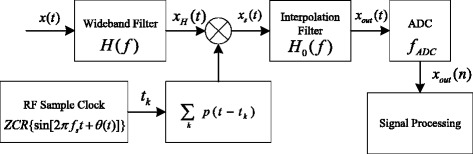
NYFR architecture

The NYFR architecture [5] is shown in Fig. 1.

In Fig. 1, The NYFR uses zero crossing rising (ZCR) voltage time to control the radio frequency (RF) sample clock and generate the RF LOS *p*(*t*) which is a non-uniform sampling LOS with a certain modulation type. As long as the modulation information of the LOS remains unchanged, we can simplify the LOS as [8]

1 $$ p(t)={\displaystyle \sum_{k=-\infty}^{\infty}\delta \left(m(t)-2\pi k\right)} $$

where *m*(*t*) = 2*πf*_*s*_*t* + *θ*_LOS_(*t*) + *φ*_LOS_, *k* is an integer, *f*_*s*_ is the LOS carrier frequency which equals the value of NZ bandwidth when the input signal is complex, define (−*f*_*s*_/2, *f*_*s*_/2) as the 0th NZ, hence, (*kf*_*s*_ − *f*_*s*_/2, *kf*_*s*_ + *f*_*s*_/2) is the *k*th NZ, *θ*_LOS_(*t*) is the LOS modulation, and *φ*_LOS_ is the LOS initial phase.

Firstly, the input analog signal *x*(*t*) is filtered by a pre-select filter *H*(*f*). Then, *x*(*t*) is mixed by the non-uniform LOS and we have *x*_*s*_(*t*) = *x*_*H*_(*t*)*p*(*t*), where *x*_*H*_(*t*) is the output of the pre-select filter. The non-uniform sampled signal *x*_*s*_(*t*) is filtered by an interpolation filter *H*_0_(*f*) with pass band (−*f*_*s*_/2, *f*_*s*_/2) and we obtain *x*_out_(*t*) which contains the LOS modulation information as the output of the NYFR [5]. Finally, *x*_out_(*t*) is sampled by the ADC whose sampling rate is *f*_ADC_ to get the discrete NYFR output.

**Fig. 2:**
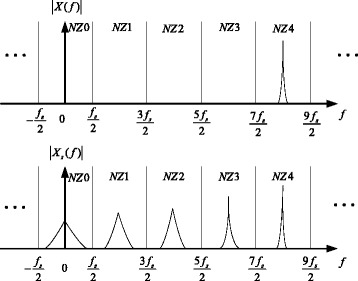
Spectra of the NYFR input signal and the non-uniform sampled signal

The input signal can be recovered by *x*_out_(*t*) and the NZ information [6]. Figure 2 illustrates the spectrum of the input signal *x*(*t*) and the spectrum of the non-uniform signal *x*_*s*_(*t*) which contains the NZ information. The NYFR output is equal to the spectrum of *x*_*s*_(*t*) in the 0th NZ after *X*_*s*_(*f*) is filtered by *H*_0_(*f*).

### LFM/BPSK signal intercepted by the NYFR

Let us denote the LFM/BPSK hybrid modulated signal as the NYFR input and it can be expressed as [2]

2 $$ x(t)=A{e}^{j2\pi {f}_ct+j\pi {\mu}_0{t}^2+j\phi (t)+j{\varphi}_0} $$

where *t* ∈ [0, *T*), *T* is the signal duration, *f*_*c*_ is the signal carrier frequency, *μ*_0_ is the chirp rate, *ϕ*(*t*) is the BPSK modulation and its value is 0 or *π*, and *φ*_0_ is the initial phase.

According to [5], sinusoidal frequency modulation (SFM) is selected as the NYFR LOS modulation, which means *m*(*t*) = 2*πf*_*s*_*t* + *m*_*f*_ sin(2*πf*_sin_*t*) + *φ*_LOS_ in (1), where *m*_*f*_ is the modulation coefficient, *f*_sin_ is the modulation frequency, and *φ*_LOS_ is the LOS initial phase. Considering the LFM/BPSK signal in (2), the output signal of the interpolation filter *H*_0_(*f*) in Fig. 1 can be expressed as [8]

3 $$ {x}_{\mathrm{out}}(t)=A{e}^{j2\pi \left({f}_c-{k}_{\mathrm{NZ}}{f}_s\right)t+j\pi {\mu}_0{t}^2+j\phi (t)-j{k}_{\mathrm{NZ}}{m}_f \sin \left(2\pi {f}_{\sin }t\right)+j{\varphi}_0-j{k}_{\mathrm{NZ}}{\varphi}_{\mathrm{LOS}}}+w(t) $$

where *k*_NZ_ is the NZ index which can indicate the original carrier frequency of the input signal [5], *t* ∈ [0, *T*), *T* is the signal duration, and *w*(*t*) is the additive white Gaussian noise [8].

From (3), the NYFR output signal contains three modulations (i.e., LFM/BPSK/SFM), and it turns to be more complex compared with the input signal (i.e., LFM/BPSK). Nevertheless, for the SFM modulation part in (3), the only unknown parameter is the NZ index *k*_NZ_. For the LFM/BPSK signal intercepted by a non-cooperative radar signal receiver, the main parameters that need to be estimated are the chirp rate, the carrier frequency, and the code length. Besides, the code length can be calculated by the positions of the phase change points. Thus, the chirp rate, the NZ index, the carrier frequency, and the code length in (3) are the parameters needed to be estimated in this paper. To simplify the following derivation, the initial phase in (3) is omitted.

The ADC sampling rate *f*_ADC_ in Fig. 1 satisfies the Nyquist sampling theorem and the sampling interval is *T*_ADC_ = 1/*f*_ADC_, the number of the total sampling points can be computed as *N* = *f*_ADC_*T*. Hence, the discrete expression of (3) is

4 $$ {x}_{\mathrm{out}}\left(n{T}_{\mathrm{ADC}}\right)=A{e}^{j2\pi \left({f}_c-{k}_{\mathrm{NZ}}{f}_s\right)\left(n{T}_{\mathrm{ADC}}\right)+j\pi {\mu}_0{\left(n{T}_{\mathrm{ADC}}\right)}^2+j\phi \left(n{T}_{\mathrm{ADC}}\right)-j{k}_{\mathrm{NZ}}{m}_f \sin \left(2\pi {f}_{\sin}\left(n{T}_{\mathrm{ADC}}\right)\right)}+w\left(n{T}_{\mathrm{ADC}}\right) $$

where *n* = 0, ⋯ *N* − 1.

## NYFR output signal parameter estimation

For the NYFR output signal in (4), it contains three modulations (i.e., LFM, BPSK, and SFM). Normally, the time-frequency transform is employed to extract the signal characteristics for a non-stationary signal. Because the NYFR output in our paper contains three modulations, some time-frequency transform methods cannot achieve an optimal result. For instance, the modulations of BPSK and SFM in the NYFR output signal cannot be extracted properly by using fractional Fourier transform [14] which is suitable for the LFM modulation. Meanwhile, ZAM works well for the BPSK modulation [12], but it is poor for the LFM modulation [13]. In addition, the time-frequency representation of (4) is no longer a straight line, which may lead to the polynomial curve fitting method [12] failing to estimate the chirp rate. Therefore, it may be difficult to find a time-frequency kernel which is optimal for the three modulations simultaneously. In this paper, we will focus on the self-characteristic and the prior information of the NYFR output signal to estimate the parameters in (4) instead of the time-frequency transformation method.

### Chirp rate estimation based on CSVR spectrum

As to the NYFR output signal parameter estimation steps, the existing algorithm constructs a multi-channel architecture to remove the LOS modulation by estimating the NZ index through extracting frequency domain feature for each channel firstly and then estimates other parameters using conventional methods [8, 11]. This algorithm regards the LOS modulation information as a redundant part and neglects the known information in it, which means the periodic characteristic of the LOS modulation. In addition, the accuracy of chirp rate estimation using the existing method will be affected by the NZ index estimation result. In order to improve the chirp rate estimation performance, we will estimate the chirp rate directly by using the LOS periodic information instead of estimating the NZ index firstly.

The square processing is applied to the data in (4) to eliminate the BPSK modulation, which means ${\left[{e}^{j\phi \left(n{T}_{\mathrm{ADC}}\right)}\right]}^2=1$. The carrier frequency in (4) can be written as *f*_0_ = *f*_*c*_ − *k*_NZ_*f*_*s*_ and we have

5 $$ {x}_{sq}\left(n{T}_{\mathrm{ADC}}\right)={A}^2{e}^{j2\pi 2{f}_0\left(n{T}_{\mathrm{ADC}}\right)+j\pi 2{\mu}_0{\left(n{T}_{\mathrm{ADC}}\right)}^2-j2{k}_{\mathrm{NZ}}{m}_f \sin \left(2\pi {f}_{\sin}\left(n{T}_{\mathrm{ADC}}\right)\right)}+{w}^{\prime}\left(n{T}_{\mathrm{ADC}}\right) $$

where *w*′(*nT*_ADC_) = 2*x*_out_(*nT*_ADC_)*w*(*nT*_ADC_) + *w*^2^(*nT*_ADC_) is the noise after the square processing. To simplify the following discussion, the noise part is omitted.

Because the SFM modulation part *m*_*f*_ sin(2*πf*_sin_*t*) in (5) is known, the LOS modulation period can be calculated as 1/*f*_sin_ and the number of points in one LOS modulation period is *N*_*c*_ = *f*_ADC_/*f*_sin_. In addition, for the NYFR structure, *f*_sin_ and *f*_ADC_ are the prior parameters, thus we can set *N*_*c*_ = *f*_ADC_/*f*_sin_ ∈ *Z*^+^, and *M*_*c*_ = floor(*N*/*N*_*c*_), where floor(⋅) means choosing the integer part of *N*/*N*_*c*_, *M*_*c*_ ∈ *Z*^+^, and *M*_*c*_ < *N*_*c*_. The above setting implies the number of signal points we use in this section is *M*_*c*_*N*_*c*_, and if the input data length *N* > *M*_*c*_*N*_*c*_, we select *M*_*c*_*N*_*c*_ points and omit the remaining points. According to the LOS periodic characteristic, we can model the data in (5) as an *M*_*c*_ × *N*_*c*_ matrix

6 $$ {\mathbf{X}}_{\mathbf{c}}=\left[\begin{array}{ccc}\hfill {x}_{sq}(0)\hfill & \hfill \cdots \hfill & \hfill {x}_{sq}\left({N}_c-1\right)\hfill \\ {}\hfill {x}_{sq}\left({N}_c\right)\hfill & \hfill \cdots \hfill & \hfill {x}_{sq}\left(2{N}_c-1\right)\hfill \\ {}\hfill \vdots \hfill & \hfill \ddots \hfill & \hfill \vdots \hfill \\ {}\hfill {x}_{sq}\left(\left({M}_c-1\right){N}_c\right)\hfill & \hfill \cdots \hfill & \hfill {x}_{sq}\left({M}_c{N}_c-1\right)\hfill \end{array}\right] $$

The relationship between the elements in the *p*th row and the *q*th row in **X**_**c**_ can be calculated as

7 $$ \begin{array}{c}\hfill \frac{x_{sq}\left(p{N}_c+n\right)}{x_{sq}\left(q{N}_c+n\right)}=\frac{A^2{e}^{j2\pi 2{f}_0\left(\left(p{N}_c+n\right){T}_{\mathrm{ADC}}\right)+j\pi 2{\mu}_0{\left(\left(p{N}_c+n\right){T}_{\mathrm{ADC}}\right)}^2-j2{k}_{\mathrm{NZ}}{m}_f \sin \left(2\pi {f}_{\sin}\left(\left(p{N}_c+n\right){T}_{\mathrm{ADC}}\right)\right)}}{A^2{e}^{j2\pi 2{f}_0\left(\left(q{N}_c+n\right){T}_{\mathrm{ADC}}\right)+j\pi 2{\mu}_0{\left(\left(q{N}_c+n\right){T}_{\mathrm{ADC}}\right)}^2-j2{k}_{\mathrm{NZ}}{m}_f \sin \left(2\pi {f}_{\sin}\left(\left(q{N}_c+n\right){T}_{\mathrm{ADC}}\right)\right)}}\hfill \\ {}\hfill ={e}^{j2\pi 2{f}_0\left(p{N}_c-q{N}_c\right){T}_{\mathrm{ADC}}+j\pi 2{\mu}_0\left[{\left(p{N}_c+n\right)}^2{T}_{\mathrm{ADC}}^2-{\left(q{N}_c+n\right)}^2{T}_{\mathrm{ADC}}^2\right]+j2{k}_{\mathrm{NZ}}{m}_f\left\{ \sin \left[2\pi {f}_{\sin}\left(\left(q{N}_c+n\right){T}_{\mathrm{ADC}}\right)\right]- \sin \left[2\pi {f}_{\sin}\left(\left(p{N}_c+n\right){T}_{\mathrm{ADC}}\right)\right]\right\}}\hfill \end{array} $$

In addition, because *N*_*c*_ = *f*_ADC_/*f*_sin_, (7) can be written as

8 $$ \begin{array}{c}\hfill \frac{x_{sq}\left(p{N}_c+n\right)}{x_{sq}\left(q{N}_c+n\right)}={e}^{j2\pi 2{f}_0\left(p{N}_c-q{N}_c\right){T}_{\mathrm{ADC}}+j\pi 2{\mu}_0\left[{\left(p{N}_c+n\right)}^2{T}_{\mathrm{ADC}}^2-{\left(q{N}_c+n\right)}^2{T}_{\mathrm{ADC}}^2\right]+j2{k}_{\mathrm{NZ}}{m}_f\left[ \sin \left(2\pi {f}_{\sin }n{T}_{\mathrm{ADC}}+2\pi q\right)- \sin \left(2\pi {f}_{\sin }n{T}_{\mathrm{ADC}}+2\pi p\right)\right]}\hfill \\ {}\hfill ={e}^{j2\pi 2{f}_0\left(p{N}_c-q{N}_c\right){T}_{\mathrm{ADC}}+j\pi 2{\mu}_0\left[{\left(p{N}_c+n\right)}^2{T}_{\mathrm{ADC}}^2-{\left(q{N}_c+n\right)}^2{T}_{\mathrm{ADC}}^2\right]}\hfill \end{array} $$

From (8), it can be observed when (8) has no LFM modulation part, the quotient of the elements in the *p*th row and the *q*th row will be a constant. Therefore, we can construct a matrix

9 $$ {\mathbf{S}}_{\mathbf{LFM}}\left(\mu \right)=\left[\begin{array}{ccc}\hfill {s}_{\mathrm{LFM}}(0)\hfill & \hfill \cdots \hfill & \hfill {s}_{\mathrm{LFM}}\left({N}_c-1\right)\hfill \\ {}\hfill {s}_{\mathrm{LFM}}\left({N}_c\right)\hfill & \hfill \cdots \hfill & \hfill {s}_{\mathrm{LFM}}\left(2{N}_c-1\right)\hfill \\ {}\hfill \vdots \hfill & \hfill \ddots \hfill & \hfill \vdots \hfill \\ {}\hfill {s}_{\mathrm{LFM}}\left(\left({M}_c-1\right){N}_c\right)\hfill & \hfill \cdots \hfill & \hfill {s}_{\mathrm{LFM}}\left({M}_c{N}_c-1\right)\hfill \end{array}\right] $$

where ${s}_{\mathrm{LFM}}(n)={e}^{-j\pi 2\mu {\left(n{T}_{\mathrm{ADC}}\right)}^2}$, *μ* is an argument. Then, we have

10 $$ \mathbf{Y}\left(\mu \right)={\mathbf{S}}_{\mathbf{LFM}}\left(\mu \right)\ast {\mathbf{X}}_{\mathbf{c}} $$

where * is the Hadamard product. When *μ* = *μ*_0_, **Y**(*μ*_0_) = **S**_**LFM**_(*μ*_0_) ∗ **X**_**c**_ will become an SFM signal matrix and we call **S**_**LFM**_(*μ*_0_) as the matching matrix.

Once the constructed matrix **S**_**LFM**_(*μ*) meets the matching matrix, (10) will be a matrix whose row elements are equal to the data in one LOS modulation period. Then the singular value decomposition (SVD) of (10) can be computed as $\mathbf{Y}\left(\mu \right)={\mathbf{U}}_{\mathbf{Y}}{\boldsymbol{\Sigma}}_{\mathbf{Y}}{\mathbf{V}}_{\mathbf{Y}}^{\mathbf{H}}$ [15], where **Σ**_**Y**_ is an *M*_*c*_ × *N*_*c*_ diagonal matrix and we call it as the singular values matrix, the singular values are ${\lambda}_1,{\lambda}_2,\cdots, {\lambda}_{M_c}$ and ${\lambda}_1\ge {\lambda}_2\ge \cdots \ge {\lambda}_{M_c}$. Based on the SVD ratio (SVR) spectrum [15, 16] and the LOS periodic characteristic, we define the chirp SVR (CSVR) spectrum as

11 $$ P\left(\mu \right)=\frac{\lambda_1^2}{{\overline{\lambda}}_{\mathrm{res}}} $$

where $$ {\overline{\lambda}}_{\mathrm{res}}=\frac{\lambda_2^2+\cdots +{\lambda}_{M_c}^2}{M_c-1} $$.

Considering the noise-free situation, when *μ* = *μ*_0_, the first singular value *λ*_1_ in **Σ**_**Y**_ will achieve its maximum and the rest singular values are 0. We call *λ*_1_ is the principle singular value and other singular values are the non-principle singular values. While *μ* ≠ *μ*_0_, the periodic characteristic of the LOS in each row of **Y**(*μ*) will be disturbed by the LFM modulation, and consequently, the non-principle singular values of **Y**(*μ*) will be non-zero according to the energy conservation theory [16]. Therefore, we can search the peak of CSVR spectrum in (11) whose argument is the chirp rate and the estimated chirp rate is

$$ {\widehat{\mu}}_0=\underset{\mu }{ \arg}\left\{ max\left[P\left(\mu \right)\right]\right\} $$

One issue to note is that when *μ* is close to *μ*_0_, **Y**(*μ*) will approximate an SFM signal. In order to keep the non-periodic characteristic of LFM signal in **Y**(*μ*) when *μ* ≠ *μ*_0_, we need to guarantee that the bandwidth of LFM signal in **Y**(*μ*) is wide enough. Because the chirp rate is unknown, the longer of the signal length we use will bring the wider of the LFM signal bandwidth, which means we can get a better resolution capability for the CSVR spectrum if we use more signal data. Because we can obtain *μ*_0_ by scanning different values of *μ* and the interval value of *μ* is not limited by the data length in (5), we say the CSVR spectrum has the property of super resolution.

Considering the situation that the data in (5) contain noise, the singular values of **Y**(*μ*) will be affected by it. When *μ* = *μ*_0_, the non-principle singular values of **Y**(*μ*) will be non-zero, and when *μ* ≠ *μ*_0_, the non-principle singular values will also be affected by noise. Therefore, the purpose that we use ${\overline{\lambda}}_{\mathrm{res}}$ in (11) rather than ${\lambda}_2^2$ in [16] is to reduce the noise effect to the non-principle singular values through average operation.

Let us analyze the complexity of CSVR spectrum. Let *N*_search_ be the number of the chirp rate scanning points. For each scanning point, the flop count [17] for Hadamard product is *M*_*c*_*N*_*c*_. Because the CSVR spectrum only requires the singular values of **Y**(*μ*) and the singular vector matrix **U**_**Y**_ and **V**_**Y**_ need not be computed, the flop count for computing **Σ**_**Y**_ is $2{M}_c{N}_c^2+2{N}_c^3$ [17]. The flop count for average operation is *M*_*c*_ and the computational complexity of peak search is *N*_search_. Thus, for the proposed method, the total number of flops is ${N}_{\mathrm{search}}\left(2{M}_c{N}_c^2+2{N}_c^3+{M}_c{N}_c+{M}_c\right)$ and the computational complexity of peak search is *N*_search_. In addition, some fast SVD methods [18, 19] may enhance the computational speed.

Then, let us compare the computational complexity of chirp rate estimation using an existing method [8]. Firstly, the existing method requires constructing *L* channels and the flop count for constructing the channel needs *NL* multiplications. Then, for each channel, it needs fast Fourier transform whose flop number is $\frac{N}{2}{ \log}_2(N)$, instantaneous auto-correlation whose flop number is *N*, and peak search whose computational complexity is *N*. The computational complexity of maximum peak finding for the *L* channels is *L* and the SFM demodulation for the input signal requires *N* multiplications. Finally, the computational complexity of chirp rate estimation step requires $\frac{N}{2}{ \log}_2(N)+N$ flops and *N* search. Thus, for the existing method, the total number of flops is $L\left[N+\frac{N}{2}{ \log}_2(N)\right]+N+\frac{N}{2}{ \log}_2(N)+N$ and the computational complexity of peak search is *LN* + *L* + *N*.

Although the computational complexity of proposed method is larger than the existing method, the estimation accuracy of the proposed method will be better than the existing method because of the super resolution property. In addition, because the chirp rate is estimated directly in the proposed method, its estimation performance will not be affected by the NZ index estimation result. In contrast, the existing chirp rate estimation method using multi-channel structure needs NZ index estimation result and its performance will be affected by it.

### NZ index estimation based on matching component function

Once the chirp rate has been obtained, the NYFR output hybrid modulated signal can be simplified via the de-chirp method. In order to estimate the carrier frequency, we need to get the NZ index first. The de-chirp signal is assumed as ${s}_{\mathrm{dechirp}}\left(n{T}_{\mathrm{ADC}}\right)={e}^{-j\pi 2{\widehat{\mu}}_0{\left(n{T}_{\mathrm{ADC}}\right)}^2}$, *n* = {0, 1, ⋯, *M*_*c*_*N*_*c*_ − 1} and we use the data in (5) with the same length to operate the de-chirp process. Omit the noise part and we have

$$ {x}_{\mathrm{de}}\left(n{T}_{\mathrm{ADC}}\right)={x}_{sq}\left(n{T}_{\mathrm{ADC}}\right){s}_{\mathrm{de}\mathrm{chirp}}\left(n{T}_{\mathrm{ADC}}\right)={A}^2{e}^{j2\pi 2{f}_0\left(n{T}_{\mathrm{ADC}}\right)+j\pi 2\left({\mu}_0-{\widehat{\mu}}_0\right){\left(n{T}_{\mathrm{ADC}}\right)}^2-j2{k}_{\mathrm{NZ}}{m}_f \sin \left(2\pi {f}_{\sin}\left(n{T}_{\mathrm{ADC}}\right)\right)} $$

Because the CSVR spectrum has super resolution capability, the transfer error of chirp rate is small and *x*_de_(*nT*_ADC_) can be written as

$$ {x}_{\mathrm{de}}\left(n{T}_{\mathrm{ADC}}\right)={A}^2{e}^{j2\pi 2{f}_0\left(n{T}_{\mathrm{ADC}}\right)-j2{k}_{\mathrm{NZ}}{m}_f \sin \left(2\pi {f}_{\sin}\left(n{T}_{\mathrm{ADC}}\right)\right)} $$

*x*_de_(*nT*_ADC_) is an SFM signal and the unknown parameters are the NZ index *k*_NZ_ and carrier frequency *f*_0_. For the NZ index estimation, the multi-channel structure is a common method [8, 11]. This method requires Fourier transform and peak search in frequency domain for each channel. It regards the SFM modulation part as a redundancy and neglects the self-characteristic of SFM signal. Here, we will use the self-characteristic of SFM signal and propose an NZ index estimation method using matching component.

According to the LOS prior information, construct a signal

12 $$ {s}_{\mathrm{SFM}}\left(n{T}_{\mathrm{ADC}},k\right)={e}^{j2k{m}_f \sin \left(2\pi {f}_{\sin}\left(n{T}_{\mathrm{ADC}}\right)\right)} $$

where the argument *k* ∈ {0, 1, ⋯, *L* − 1} and *n* = {0, 1, ⋯, *M*_*c*_*N*_*c*_ − 1}. For *x*_de_(*nT*_ADC_), we have

$$ {y}_{\mathrm{de}}\left(n{T}_{\mathrm{ADC}},k\right)={s}_{\mathrm{SFM}}\left(n{T}_{\mathrm{ADC}},k\right){x}_{\mathrm{de}}\left(n{T}_{\mathrm{ADC}}\right)={A}^2{e}^{j2\pi 2{f}_0\left(n{T}_{\mathrm{ADC}}\right)+j2\left(k-{k}_{\mathrm{NZ}}\right){m}_f \sin \left(2\pi {f}_{\sin}\left(n{T}_{\mathrm{ADC}}\right)\right)} $$

To simplify the following derivation, denote *n* = *nT*_ADC_ and the instantaneous auto-correlation of *y*_de_(*nT*_ADC_, *k*) is

13 $$ \begin{array}{c}\hfill R\left(n,k\right)={y}_{\mathrm{de}}\left(n,k\right){y}_{\mathrm{de}}^{*}\left(n+\tau, k\right)\hfill \\ {}={A}^4{e}^{-j2\pi 2{f}_0\tau }{e}^{j2\left(k-{k}_{\mathrm{NZ}}\right){m}_f\left[ \sin \left(2\pi {f}_{\sin }n\right)- \cos \left(2\pi {f}_{\sin }n\right) \sin \left(2\pi {f}_{\sin}\tau \right)- \sin \left(2\pi {f}_{\sin }n\right) \cos \left(2\pi {f}_{\sin}\tau \right)\right]}\end{array} $$

Define the matching component function as

14 $$ {P}_{\mathrm{NZ}}(k)=\left|{\displaystyle \sum_{n=0}^{N_c{M}_c-1-\tau }R\left(n,k\right)}\right| $$

According to the self-characteristic of SFM signal, we have ${e}^{jk \sin \left(2\pi {f}_0n\right)}={\displaystyle \sum_{m=-\infty}^{\infty }{J}_m(k){e}^{jm2\pi {f}_0n}}$, where *J*_*m*_(⋅) is the Bessel function with *m* order. Based on (13), (14) can be expressed as

15 $$ \begin{array}{c}\hfill {P}_{\mathrm{NZ}}(k)=\left|{A}^4{e}^{-j2\pi 2{f}_0\tau }{\displaystyle \sum_{n=0}^{N_c{M}_c-1-\tau }{\displaystyle \sum_{m_1=-\infty}^{\infty }{J}_{m_1}\left[\left(k-{k}_{\mathrm{NZ}}\right){m}_f\left(1- \cos \left(2\pi {f}_{\sin}\tau \right)\right)\right]{e}^{j{m}_12\pi {f}_{\sin }n}}{\displaystyle \sum_{m_2=-\infty}^{\infty }{J}_{m_2}\left[\left({k}_{\mathrm{NZ}}-k\right){m}_f \sin \left(2\pi {f}_{\sin}\tau \right)\right]{e}^{j{m}_22\pi {f}_{\sin }n}{e}^{j{m}_2\frac{\pi }{2}}}}\right|\hfill \\ {}\hfill =\left|{A}^4{\displaystyle \sum_{m_1=-\infty}^{\infty }{J}_{m_1}\left[\left(k-{k}_{\mathrm{NZ}}\right){m}_f\left(1- \cos \left(2\pi {f}_{\sin}\tau \right)\right)\right]}{\displaystyle \sum_{m_2=-\infty}^{\infty }{J}_{m_2}\left[\left({k}_{\mathrm{NZ}}-k\right){m}_f \sin \left(2\pi {f}_{\sin}\tau \right)\right]{e}^{j{m}_2\frac{\pi }{2}}}{\displaystyle \sum_{n=0}^{N_c{M}_c-1}{e}^{j2\pi {f}_{\sin}\left({m}_1+{m}_2\right)n}}\right|\hfill \end{array} $$

In (15), when *m*_1_ + *m*_2_ = 0, we have ${\displaystyle \sum_{n=0}^{N_c{M}_c-1-\tau }{e}^{j2\pi {f}_{\sin}\left({m}_1+{m}_2\right)n}}={N}_c{M}_c-\tau$, and when *m*_1_ + *m*_2_ ≠ 0, considering *m*_1_ + *m*_2_ ∈ *Z*^+^, *n* = *nT*_ADC_, and *M*_*c*_ ∈ *Z*^+^, we have $\left|{\displaystyle \sum_{n=0}^{N_c{M}_c-1-\tau }{e}^{j2\pi {f}_{\sin}\left({m}_1+{m}_2\right)n}}\right|=0$. Hence, (15) can be written as

${P}_{\mathrm{NZ}}(k)=\left|{A}^4\left({N}_c{M}_c-\tau \right){\displaystyle \sum_{m_1=-\infty}^{\infty }{J}_{m_1}\left[\left(k-{k}_{\mathrm{NZ}}\right){m}_f\left(1- \cos \left(2\pi {f}_{\sin}\tau \right)\right)\right]}{J}_{-{m}_1}\left[\left({k}_{\mathrm{NZ}}-k\right){m}_f \sin \left(2\pi {f}_{\sin}\tau \right)\right]{e}^{-j{m}_1\frac{\pi }{2}}\right|$_._ On the basis of the property of Bessel function $$ \left\{\begin{array}{l}{J}_m\left(-k\right)={\left(-1\right)}^m{J}_m(k)\\ {}{J}_{-m}(k)=-{J}_m(k),\kern1em m\kern1em \mathrm{is}\kern1em \mathrm{odd}\\ {}{J}_{-m}(k)={J}_m(k),\kern1em m\kern1em \mathrm{is}\kern1em \mathrm{even}\end{array}\right. $$, we have

16 $$ \begin{array}{c}\hfill {P}_{\mathrm{NZ}}(k)={A}^4\left({N}_c{M}_c-\tau \right)\left|{\displaystyle \sum_{m_1=-\infty}^{\infty}\Big\{{J}_{2{m}_1}\left[\left(k-{k}_{\mathrm{NZ}}\right){m}_f\left(1- \cos \left(2\pi {f}_{\sin}\tau \right)\right)\right]{J}_{2{m}_1}\left[\left(k-{k}_{\mathrm{NZ}}\right){m}_f \sin \left(2\pi {f}_{\sin}\tau \right)\right]{e}^{-j{m}_1\pi }}\right.\hfill \\ {}\hfill \left.+{J}_{2{m}_1+1}\left[\left(k-{k}_{\mathrm{NZ}}\right){m}_f\left(1- \cos \left(2\pi {f}_{\sin}\tau \right)\right)\right]{J}_{2{m}_1+1}\left[\left(k-{k}_{\mathrm{NZ}}\right){m}_f \sin \left(2\pi {f}_{\sin}\tau \right)\right]{e}^{-j\left(2{m}_1+1\right)\frac{\pi }{2}}\Big\}\right|\hfill \end{array} $$

when *k* = *k*_NZ_, (16) can be expressed as

17 $$ {P}_{\mathrm{NZ}}\left({k}_{\mathrm{NZ}}\right)={A}^4\left({N}_c{M}_c-\tau \right)\left|{\displaystyle \sum_{m_1=-\infty}^{\infty }{J}_{m_1}(0)}\right|={A}^4\left({N}_c{M}_c-\tau \right) $$

when *k* ≠ *k*_NZ_, because the modulation coefficient can be set as |*m*_*f*_| ≥ 1, we have $\left|{J}_{m_1}\left[\left(k-{k}_{\mathrm{NZ}}\right){m}_f\left(1- \cos \left(2\pi {f}_{\sin}\tau \right)\right)\right]\right|\ll 1$ and $\left|{J}_{m_1}\left[\left(k-{k}_{\mathrm{NZ}}\right){m}_f \sin \left(2\pi {f}_{\sin}\tau \right)\right]\right|\ll 1$. Thus, (16) can be written as

18 $$ {P}_{\mathrm{NZ}}(k)\ll {A}^4\left({N}_c{M}_c-\tau \right) $$

From (17) and (18), when *k* = *k*_NZ_, the matching component function *P*_NZ_(*k*) will achieve its maximum and we call the constructed signal *s*_SFM_(*nT*_ADC_, *k*_NZ_) as the matching component. The peak of *P*_NZ_(*k*) indicates the NZ index estimation result.

It should be noted that in order to avoid *J*_*m*_(⋅)≡0 in (16), we should make sure 1 − cos(2*πf*_sin_*τ*) ≠ 0 and sin(2*πf*_sin_*τ*) ≠ 0 in (16). Therefore, we need to guarantee 2*πf*_sin_*T*_ADC_*τ* ≠ 2*πz*, *z* ∈ *Z*, which means *τ* ≠ *zf*_ADC_/*f*_sin_. Apparently, we should also avoid *τ* → *zf*_ADC_/*f*_sin_ to prevent 1 − cos(2*πf*_sin_*τ*) → 0 and sin(2*πf*_sin_*τ*) → 0, where → means going close to. This is the selection criterion for the value of shift length *τ*. Because the LOS modulation frequency *f*_sin_ and the sampling frequency *f*_ADC_ are known, the shift length *τ* can be set as *τ* ≠ *zf*_ADC_/*f*_sin_ and it should be far away from *zf*_ADC_/*f*_sin_, *z* ∈ *Z* to satisfy the above requirements.

Furthermore, let us consider the modulation coefficient *m*_*f*_ in (16). As we analyzed before, when *k* ≠ *k*_NZ_, we have $\left|{J}_{m_1}\left[\left(k-{k}_{\mathrm{NZ}}\right){m}_f\left(1- \cos \left(2\pi {f}_{\sin}\tau \right)\right)\right]\right|\ll 1$ and $\left|{J}_{m_1}\left[\left(k-{k}_{\mathrm{NZ}}\right){m}_f \sin \left(2\pi {f}_{\sin}\tau \right)\right]\right|\ll 1$ in (16). However, if |*m*_*f*_| is very small, according to the characteristic of Bessel function, we have $\left|{J}_{m_1}\left[\left(k-{k}_{\mathrm{NZ}}\right){m}_f\left(1- \cos \left(2\pi {f}_{\sin}\tau \right)\right)\right]\right|\to 1$ and $\left|{J}_{m_1}\left[\left(k-{k}_{\mathrm{NZ}}\right){m}_f \sin \left(2\pi {f}_{\sin}\tau \right)\right]\right|\to 1$. Hence, (18) may not be guaranteed. Therefore, in order to guarantee that the matching component function has a good performance, we should make sure that |*m*_*f*_| is not too small. This is the reason we set |*m*_*f*_| ≥ 1 above.

From the peak search of *P*_NZ_(*k*), *k* ∈ {0, 1, ⋯, *L* − 1}, the NZ index can be estimated as

$$ {\widehat{k}}_{\mathrm{NZ}}=\underset{k}{ \arg}\left\{ max\left[{P}_{\mathrm{NZ}}(k)\right]\right\} $$

The flop count of the proposed method for instantaneous auto-correlation and summation are *M*_*c*_*N*_*c*_ and *M*_*c*_*N*_*c*_ − *τ*, respectively. In addition, the NZ index estimation needs to search *L* points to find the peak. Hence, for the proposed method, the total number of flops is 2*M*_*c*_*N*_*c*_ + *M*_*c*_*N*_*c*_ − *τ* and the computational complexity of peak search is *L*.

As to the method in [8], from the analysis in Section 3.1, the total number of flops is $L\left[N+\frac{N}{2}{ \log}_2(N)\right]$ and the computational complexity of peak search is *LN* + *L*. Because *M*_*c*_*N*_*c*_ ≤ *N* and *L* ≪ *N*, the proposed method has a smaller computational complexity.

According to the LOS information and the estimated NZ index ${\widehat{k}}_{\mathrm{NZ}}$, the LOS modulation in (11) can be demodulated and (11) will become a single carrier signal. Using Fourier transform to estimate the carrier frequency and we can obtain the result $2{\widehat{f}}_0$. Hence, the carrier frequency of the input LFM/BPSK signal can be calculated as ${\widehat{f}}_c={\widehat{f}}_0+{\widehat{k}}_{\mathrm{NZ}}{f}_s$.

### Phase change point estimation based on matching code and subspace

For the BPSK modulation, we not only need to estimate the code length, but also want to obtain the position of each phase change point. This section will present a phase change point estimation method for the BPSK modulation with high accuracy using matching code and subspace orthogonal property.

The chirp rate ${\widehat{\mu}}_0$ and the NZ index ${\widehat{k}}_{\mathrm{NZ}}$ have been already estimated. Let us reconsider the data in (4) and construct a signal

$$ {x}_{\mathrm{re}}\left(n{T}_{\mathrm{ADC}}\right)=A{e}^{-j\pi {\widehat{\mu}}_0{\left(n{T}_{\mathrm{ADC}}\right)}^2+j{\widehat{k}}_{\mathrm{NZ}}{m}_f \sin \left(2\pi {f}_{\sin}\left(n{T}_{\mathrm{ADC}}\right)\right)} $$

For (4), we have

19 $$ {x}_B\left(n{T}_{\mathrm{ADC}}\right)={x}_{\mathrm{out}}\left(n{T}_{\mathrm{ADC}}\right){x}_{\mathrm{re}}\left(n{T}_{\mathrm{ADC}}\right)=A{e}^{j2\pi {f}_0\left(n{T}_{\mathrm{ADC}}\right)+j\phi \left(n{T}_{\mathrm{ADC}}\right)+j2\pi \varDelta {\mu}_0{\left(n{T}_{\mathrm{ADC}}\right)}^2}+{w}^{\hbox{'}}\left(n{T}_{\mathrm{ADC}}\right) $$

where $\varDelta {\mu}_0={\mu}_0-{\widehat{\mu}}_0$ is the transfer error and *w*′(*nT*_ADC_) is the noise part. Because the NZ index estimation result is an integer, we can assume ${\widehat{k}}_{\mathrm{NZ}}={k}_{\mathrm{NZ}}$ and it has no transfer error. Since the carrier frequency ${\widehat{f}}_c$ of the NYFR input signal has been obtained, the estimation of the carrier frequency in (19) can be computed as ${\widehat{f}}_0={\widehat{f}}_c-{\widehat{k}}_{\mathrm{NZ}}{f}_s$.

Generally, we can denote *x*_*B*_(*n*) = *x*_*B*_(*nT*_ADC_). We redefine *n* = 1, ⋯ *N* and omit the initial phase. The data in (19) can be separated into several segments and the length of each segment is *N*_*s*_ which is shorter than the points of one code length. The method in [20] can be employed to obtain the coarse estimation of the code length and determine the segment length *N*_*s*_. However, this method can only give the code length estimation and it has no capability to give the position of each phase change point. The number of the data segments can be calculated as Num = floor(*N*/*N*_*s*_). We redefine *p* = 0, 1, …, Num − 1 and the signal data in the (*p* + 1)th segment can be written as

$$ {\mathbf{x}}_{{\mathbf{N}}_{\mathbf{s}}}(n)={\left[{x}_B\left(n+p{N}_s+1\right),{x}_B\left(n+p{N}_s+2\right),\dots, {x}_B\left(n+p{N}_s+{N}_s\right)\right]}^T $$

$$ \begin{array}{l}={\left[{e}^{j\phi \left(n+p{N}_s+1\right)}{e}^{j2\pi {f}_0\left(n+p{N}_s+1\right)+j\pi \varDelta {\mu}_0{\left(n+p{N}_s+1\right)}^2},\dots, {e}^{j\phi \left(n+p{N}_s+{N}_s\right)}{e}^{j2\pi {f}_0\left(n+p{N}_s+{N}_s\right)+j\pi \varDelta {\mu}_0{\left(n+p{N}_s+{N}_s\right)}^2}\right]}^T\\ {}+{\left[{w}^{\hbox{'}}\left(n+p{N}_s+1\right),\dots, {w}^{\hbox{'}}\left(n+p{N}_s+{N}_s\right)\right]}^T\end{array} $$

Rewrite ${\mathbf{x}}_{{\mathbf{N}}_{\mathbf{s}}}(n)$ and we have

20 $$ \begin{array}{c}\hfill {\mathbf{x}}_{{\mathbf{N}}_{\mathbf{s}}}(n)=\left[\begin{array}{cccc}\hfill {e}^{j\phi \left(n+p{N}_s+1\right)}\hfill & \hfill 0\hfill & \hfill \dots \hfill & \hfill 0\hfill \\ {}\hfill 0\hfill & \hfill {e}^{j\phi \left(n+p{N}_s+2\right)}\hfill & \hfill \dots \hfill & \hfill 0\hfill \\ {}\hfill \vdots \hfill & \hfill \vdots \hfill & \hfill \ddots \hfill & \hfill \vdots \hfill \\ {}\hfill 0\hfill & \hfill 0\hfill & \hfill \dots \hfill & \hfill {e}^{j\phi \left(n+p{N}_s+{N}_s\right)}\hfill \end{array}\right]\left[\begin{array}{c}\hfill {e}^{j2\pi {f}_0\left(n+p{N}_s+1\right)+j\pi \varDelta {\mu}_0{\left(n+p{N}_s+1\right)}^2}\hfill \\ {}\hfill {e}^{j2\pi {f}_0\left(n+p{N}_s+2\right)+j\pi \varDelta {\mu}_0{\left(n+p{N}_s+2\right)}^2}\hfill \\ {}\hfill \vdots \hfill \\ {}\hfill {e}^{j2\pi {f}_0\left(n+p{N}_s+{N}_s-1\right)+j\pi \varDelta {\mu}_0{\left(n+p{N}_s+{N}_s\right)}^2}\hfill \end{array}\right]+{\mathbf{w}}^{\mathbf{\hbox{'}}}\hfill \\ {}\hfill ={\mathbf{D}}_{\mathbf{B}}\left[\begin{array}{c}\hfill {e}^{j2\pi {f}_0\left(n+p{N}_s+1\right)+j\pi \varDelta {\mu}_0{\left(n+p{N}_s+1\right)}^2}\hfill \\ {}\hfill {e}^{j2\pi {f}_0\left(n+p{N}_s+2\right)+j\pi \varDelta {\mu}_0{\left(n+p{N}_s+2\right)}^2}\hfill \\ {}\hfill \vdots \hfill \\ {}\hfill {e}^{j2\pi {f}_0\left(n+p{N}_s+{N}_s\right)+j\pi \varDelta {\mu}_0{\left(n+p{N}_s+{N}_s\right)}^2}\hfill \end{array}\right]+{\mathbf{w}}^{\mathbf{\hbox{'}}}\hfill \end{array} $$

where **D**_**B**_ is the BPSK modulation matrix. If there is no phase change point in the BPSK modulation matrix, **D**_**B**_ will become a unit matrix. In this paper, we mark the unit matrix as **I**.

A BPSK modulation matrix $\mathbf{D}\left({k}_s\right)=\mathrm{diag}\left[{e}^{j{\theta}_1},{e}^{j{\theta}_2},\dots, {e}^{j{\theta}_i},\dots, {e}^{j{\theta}_{N_s}}\right]$ can be constructed, where *i* = 1, …, *N*_*s*_ and $$ {\theta}_i=\left\{\begin{array}{l}0\kern1em i<{k}_s\\ {}\pi \kern1em i\ge {k}_s\kern0.5em \end{array}\right. $$, 1 ≤ *k*_*s*_ ≤ *N*_*s*_, which implies that the phase change point in **D**(*k*_*s*_) will be presented point by point at *i* = *k*_*s*_ with the moving of *k*_*s*_. To estimate the phase change point in one segment, two situations should be taken into consideration:

1. The data in one segment contain one phase change point, which means **D**_**B**_ in (20) contains one phase change point.
  Under this circumstance, the position of the phase change point in **D**_**B**_ is assumed as *k*_phase_, 1 < *k*_phase_ < *N*_*s*_. When *k*_*s*_ = *k*_phase_ in **D**(*k*_*s*_), **D**(*k*_phase_)**D**_**B**_ = ± **I** and we call **D**(*k*_*s*_) is the matching code of **D**_**B**_. Meanwhile, when *k*_*s*_ ≠ *k*_phase_, we have **D**(*k*_phase_)**D**_**B**_ ≠ ± **I**.
2. The data in one segment have no phase change point, which means **D**_**B**_ has no phase change point.

Under this condition, when *k*_*s*_ = 1 or *k*_*s*_ = *N*_*s*_, we have **D**(*k*_*s*_)**D**_**B**_ = ± **I**, which implies **D**(*k*_*s*_) will match **D**_**B**_ when *k*_*s*_ is at the edge of the data segment. When *k*_*s*_ = *k*_phase_ = 1 or *k*_*s*_ = *k*_phase_ = *N*_*s*_, we also mark **D**(*k*_*s*_) as the matching code **D**(*k*_phase_).

Therefore, combining (20) and **D**(*k*_phase_) yield

$$ \mathbf{D}\left({k}_{\mathrm{phase}}\right){\mathbf{x}}_{{\mathbf{N}}_{\mathbf{s}}}(n)=\pm \left[\begin{array}{c}\hfill {e}^{j2\pi {f}_0\left(n+p{N}_s+1\right)+j\pi \varDelta {\mu}_0{\left(n+p{N}_s+1\right)}^2}\hfill \\ {}\hfill {e}^{j2\pi {f}_0\left(n+p{N}_s+2\right)+j\pi \varDelta {\mu}_0{\left(n+p{N}_s+2\right)}^2}\hfill \\ {}\hfill \vdots \hfill \\ {}\hfill {e}^{j2\pi {f}_0\left(n+p{N}_s+{N}_s\right)+j\pi \varDelta {\mu}_0{\left(n+p{N}_s+{N}_s\right)}^2}\hfill \end{array}\right]+{\mathbf{w}}^{\mathbf{{\prime\prime}}} $$

where **w**″ is the noise part. The above equation can be rewritten as

21 $$ \mathbf{D}\left({k}_{\mathrm{phase}}\right){\mathbf{x}}_{{\mathbf{N}}_{\mathbf{s}}}(n)={\mathbf{D}}_{\boldsymbol{\Delta} {\boldsymbol{\upmu}}_{\mathbf{0}}}\mathbf{A}\left({f}_0\right){e}^{j2\pi {f}_0\left(n+p{N}_s+1\right)}+{\mathbf{w}}^{\mathbf{{\prime\prime}}} $$

where the chirp rate transfer error matrix is

$$ {\mathbf{D}}_{\boldsymbol{\Delta} {\boldsymbol{\upmu}}_{\mathbf{0}}}=\mathrm{diag}\left[{e}^{j\pi \varDelta {\mu}_0{\left(n+p{N}_s+1\right)}^2},{e}^{j\pi \varDelta {\mu}_0{\left(n+p{N}_s+2\right)}^2},\dots, {e}^{j\pi \varDelta {\mu}_0{\left(n+p{N}_s+{N}_s\right)}^2}\right] $$

and the driving vector is

$$ \mathbf{A}\left({f}_0\right)=\pm {\left[1,{e}^{j2\pi {f}_0},\dots, {e}^{j2\pi {f}_0\left({N}_s-1\right)}\right]}^T $$

Because the carrier frequency ${\widehat{f}}_0$ has been already estimated, we can construct a signal ${s}_{{\widehat{f}}_0}(n)={e}^{j2\pi {\widehat{f}}_0n}$ whose data length is *N*_*s*_. Then, we have

22 $$ {\mathbf{s}}_{{\widehat{\mathbf{f}}}_{\mathbf{0}}}(n)={\left[{s}_{{\widehat{f}}_0}\left(n+1\right),{s}_{{\widehat{f}}_0}\left(n+2\right),\dots, {s}_{{\widehat{f}}_0}\left(n+{N}_s\right)\right]}^T={\left[1,{e}^{j2\pi {\widehat{f}}_0},\dots, {e}^{j2\pi {\widehat{f}}_0\left({N}_s-1\right)}\right]}^T{e}^{j2\pi {\widehat{f}}_0\left(n+1\right)} $$

The auto-correlation of ${\mathbf{s}}_{{\widehat{\mathbf{f}}}_{\mathbf{0}}}(n)$ is ${\mathbf{R}}_{\mathbf{s}\mathbf{s}}=E\left[{\mathbf{s}}_{{\widehat{\mathbf{f}}}_{\mathbf{0}}}(n){\mathbf{s}}_{{\widehat{\mathbf{f}}}_{\mathbf{0}}}^{\mathbf{H}}(n)\right]$ and the rank of **R**_**ss**_ is rank(**R**_**ss**_) = 1. Hence, the noise subspace of **R**_**ss**_ can be computed and it can be denoted as **G**.

Firstly, let us consider (21) is noise free. According to the noise subspace **G** from ${\mathbf{s}}_{{\widehat{\mathbf{f}}}_{\mathbf{0}}}(n)$, we get

23 $$ {\left[\mathbf{D}\left({k}_{\mathrm{phase}}\right){\mathbf{x}}_{{\mathbf{N}}_{\mathbf{s}}}(n)\right]}^{\mathbf{H}}\mathbf{G}={e}^{-j2\pi {f}_0\left(n+p{N}_s+1\right)}{\mathbf{A}}^{\mathbf{H}}\left({f}_0\right){\mathbf{D}}_{\boldsymbol{\Delta} {\boldsymbol{\upmu}}_{\mathbf{0}}}^{\mathbf{H}}\mathbf{G} $$

The result of (23) will be affected by the chirp rate transfer error *Δμ*_0_ and the carrier frequency transfer error ${f}_0-{\widehat{f}}_0$. If *Δμ*_0_ = 0 and ${f}_0={\widehat{f}}_0$, we have ${\mathbf{D}}_{\boldsymbol{\Delta} {\boldsymbol{\upmu}}_{\mathbf{0}}}^{\mathbf{H}}=\mathbf{I}$ and **A**^**H**^(*f*_0_)**G** = **0**, where **I** is an *N*_*s*_ × *N*_*s*_ unit matrix and **0** is a 1 × (*N*_*s*_ − 1) zero vector. Thus, the result of (23) can be calculated as

24 $$ {\left[\mathbf{D}\left({k}_{\mathrm{phase}}\right){\mathbf{x}}_{{\mathbf{N}}_{\mathbf{s}}}(n)\right]}^{\mathbf{H}}\mathbf{G}={e}^{-j2\pi {f}_0\left(n+p{N}_s+1\right)}{\mathbf{A}}^{\mathbf{H}}\left({f}_0\right)\mathbf{G}=\mathbf{0} $$

Then, we can define the phase search pseudo-spectrum as

25 $$ \mathrm{Phase}\left({k}_s\right)=\frac{1}{{\left[\mathbf{D}\left({k}_s\right){\mathbf{x}}_{{\mathbf{N}}_{\mathbf{s}}}(n)\right]}^{\mathbf{H}}\mathbf{G}{\mathbf{G}}^{\mathbf{H}}\left[\mathbf{D}\left({k}_s\right){\mathbf{x}}_{{\mathbf{N}}_{\mathbf{s}}}(n)\right]} $$

where 1 ≤ *k*_*s*_ ≤ *N*_*s*_.

When *k*_*s*_ = *k*_phase_ (i.e., **D**(*k*_*s*_) matches **D**_**B**_), (25) will achieve its maximum and the corresponding peak position ${\widehat{k}}_s$ can be calculated by

26 $$ {\widehat{k}}_s=\underset{k_s}{ \arg}\left\{ \max \frac{1}{{\left[\mathbf{D}\left({k}_s\right){\mathbf{x}}_{{\mathbf{N}}_{\mathbf{s}}}(n)\right]}^{\mathbf{H}}\mathbf{G}{\mathbf{G}}^{\mathbf{H}}\left[\mathbf{D}\left({k}_s\right){\mathbf{x}}_{{\mathbf{N}}_{\mathbf{s}}}(n)\right]}\right\} $$

When Phase(*k*_*s*_) reaches its maximum, $\mathbf{D}\left({\widehat{k}}_s\right)$ will become the matching code and the corresponding point ${\widehat{k}}_s$ is the estimated position of the phase change point in one data segment. If the data in one segment have no phase change point, Phase(*k*_*s*_) will show a peak at the first or the last point of the data segment from the analysis of situation (2).

According to (24) and (25), if the transfer errors of chirp rate and carrier frequency are 0, the width of the peak in (25) will be one point. Thus, the phase search pseudo-spectrum can obtain an accurate estimation result and its peak width is independent of the data segment length comparing with the wavelet transform method whose peak width is decided by the length of the scale [19].

However, if the chirp rate transfer error *Δμ*_0_ is not 0, the transfer error matrix of chirp rate will be no longer a unit matrix. With the increasing of *Δμ*_0_, ${\mathbf{D}}_{\boldsymbol{\Delta} {\boldsymbol{\upmu}}_{\mathbf{0}}}$ will be far away from a unit matrix. Meanwhile, (24) will also be far away from **0** and the width of the peak in (25) will be expanded. In addition, if the carrier frequency transfer error is not 0, the orthogonal relation between **A**^**H**^(*f*_0_) and **G** in (24) will not be satisfied. Thus, the width of the peak in (25) will also be expanded. In summary, the transfer error from chirp rate and carrier frequency will deteriorate the accuracy of phase change point estimation.

Fortunately, because the CSVR spectrum is a super resolution method, the chirp rate transfer error *Δμ*_0_ → 0. In addition, because the NZ index estimation result is an integer and it has no transfer error, we have ${\widehat{f}}_0\to {f}_0$. Hence, (24) can be written as

27 $$ {\left[\mathbf{D}\left({k}_{\mathrm{phase}}\right){\mathbf{x}}_{{\mathbf{N}}_{\mathbf{s}}}(n)\right]}^{\mathbf{H}}\mathbf{G}\to \mathbf{0} $$

Then, let us consider the signal ${\mathbf{x}}_{{\mathbf{N}}_{\mathbf{w}}}(n)$ containing noise. According to (23) and (27), if **D**(*k*_*s*_) matches **D**_**B**_, we get

$$ {\left[\mathbf{D}\left({k}_{\mathrm{phase}}\right){\mathbf{x}}_{{\mathbf{N}}_{\mathbf{s}}}(n)\right]}^{\mathbf{H}}\mathbf{G}\to \mathbf{0}+{{\mathbf{w}}^{\mathbf{{\prime\prime}}}}^{\mathbf{H}}\mathbf{G} $$

If **D**(*k*_*s*_) does not match **D**_**B**_, we have

$$ {\left[\mathbf{D}\left({k}_s\right){\mathbf{x}}_{{\mathbf{N}}_{\mathbf{s}}}(n)\right]}^{\mathbf{H}}\mathbf{G}\to {\mathbf{x}}_{{\mathbf{N}}_{\mathbf{s}}}^{\mathbf{H}}(n){\mathbf{D}}^{\mathbf{H}}\left({k}_s\right)\mathbf{G}+{{\mathbf{w}}^{\mathbf{{\prime\prime}}}}^{\mathbf{H}}\mathbf{G} $$

Thus, under the condition of noised signal, the phase search pseudo-spectrum in (25) can still achieve its peak when **D**(*k*_*s*_) matches **D**_**B**_.

**Fig. 3:**
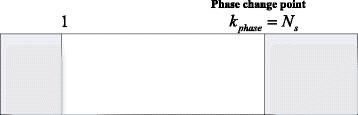
Phase change point locates at the edge of the data segment

After the peak search process is completed in one data segment, there is one issue to be considered. The position of the phase change point *k*_phase_ may locate at the first or the last point of the data segment. This situation is shown in Fig. 3.

For this condition, the following process can handle this issue.

For the (*p* + 1)th data segment, when the phase search pseudo-spectrum shows a peak at ${\widehat{k}}_s=p{N}_s+1$ or ${\widehat{k}}_s=p{N}_s+{N}_s$, we can assume *c* is a small integer, *c* > 1, and reselect the data from *pN*_*s*_ + 1 + *c* to *pN*_*s*_ + *N*_*s*_ + *c*. The reselected data can be written as

28 $$ {\tilde{\mathbf{x}}}_{{\mathbf{N}}_{\mathbf{s}}}(n)={\left[{x}_B\left(n+p{N}_s+1+c\right),{x}_B\left(n+p{N}_s+2+c\right),\dots, {x}_B\left(n+p{N}_s+{N}_s+c\right)\right]}^T $$

The result of (26) can be recalculated using the data from (28). The peak position of the phase search pseudo-spectrum using the data in (28) is denoted as ${\widehat{k}}_s^{(ag)}$ and we have

29 $$ {\widehat{k}}_s^{(ag)}=\underset{k_s}{ \arg}\left\{ \max \frac{1}{{\left[\mathbf{D}\left({k}_s\right){\tilde{\mathbf{x}}}_{{\mathbf{N}}_{\mathbf{s}}}(n)\right]}^{\mathbf{H}}\mathbf{G}{\mathbf{G}}^{\mathbf{H}}\left[\mathbf{D}\left({k}_s\right){\tilde{\mathbf{x}}}_{{\mathbf{N}}_{\mathbf{s}}}(n)\right]}\right\} $$

**Fig. 4:**
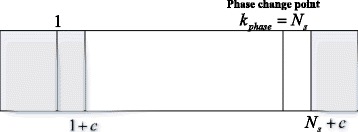
Reselection and recalculation process

This process is illustrated in Fig. 4.

If the reselected data segment shows a peak at the first point (i.e., ${\widehat{k}}_s^{(ag)}=1$), let ${\widehat{k}}_s^{(ag)}={N}_s$, which means if the data in (28) have no phase change point, the position of the peak is always at the end of this segment.

If the distance between ${\widehat{k}}_s^{(ag)}$ and the last point of the reselected data segment is not equal to *c*, we regard that the last point of the (*p* + 1)th data segment is not the phase change point. Otherwise, the last point of the (*p* + 1)th data segment covers the phase change point. For the first point of the next data segment, if the distance between ${\widehat{k}}_s^{(ag)}$ and the last point of the reselected data segment is not equal to *c* − 1, we regard that the first point of the (*p* + 2)th data segment is not the phase change point. Otherwise, the first point of the (*p* + 2)th data segment covers the phase change point.

Finally, let us compare the computational complexity of our method with the method using the Haar wavelet transform [21]. We focus on the data in one segment. The number of the points in one segment is still assumed as *N*_*s*_. Besides, we also assume that the wavelet scale contains *N*_*s*_ points. Because the noise subspace in (25) is fixed, we consider the computational complexity of matrix multiplication in one data segment. Thus, the computational complexity of the proposed method needs *N*_*s*_[*N*_*s*_ + 4*N*_*s*_(*N*_*s*_ − 1) + 2(*N*_*s*_ − 1)] number of flops in one data segment. For the method in [21], the computational complexity needs $2{N}_s^2$ number of flops for one wavelet scale. Although the proposed method requires a larger computational complexity, its estimation result can achieve a higher accuracy. Since the phase change point in one data segment has been estimated, the next section will give the code length estimation process.

## Algorithm steps

For the LFM/BPSK hybrid modulated signal intercepted by the NYFR in (4), the proposed parameter estimation algorithm steps are as follows:

1. For the NYFR output signal in (4), the square method is employed and the signal data whose length is *M*_*c*_*N*_*c*_ points are selected as shown in (5). Model the data in (5) as the matrix **X**_**c**_ which is shown in (6) based on the periodic characteristic of the LOS modulation and construct the LFM matching matrix **S**_**LFM**_(*μ*) expressed in (9). Estimate the chirp rate ${\widehat{\mu}}_0$ by computing the CSVR spectrum based on (11);
2. The de-chirp process using ${\widehat{\mu}}_0$ can be operated to eliminate the LFM modulation of the signal in (5). Construct the matching component as shown in (12). Based on the matching component function *P*_NZ_(*k*) in (14), the NZ index estimation result ${\widehat{k}}_{\mathrm{NZ}}$ can be obtained. According to the LOS information and ${\widehat{k}}_{\mathrm{NZ}}$, the NYFR output carrier frequency ${\widehat{f}}_0$ and the input hybrid modulated signal carrier frequency *f*_*c*_ can be estimated;
3. Construct the signal in (22) using ${\widehat{f}}_0$ and calculate its noise subspace **G**. Demodulate the signal in (4) using the estimated ${\widehat{\mu}}_0$ and ${\widehat{k}}_{\mathrm{NZ}}$. The data in (20) can be obtained by dividing the demodulated signal into Num segments and set *p* = 0.
4. For the data from *pN*_*s*_ + 1 to *pN*_*s*_ + *N*_*s*_ in the (*p* + 1)th data segment, calculate the phase search pseudo-spectrum in (25) using **G** and the constructed **D**(*k*_*s*_). Find the peak position ${\widehat{k}}_{s,p}$ in the (*p* + 1)th data segment. If ${\widehat{k}}_{s,p}\in \left(p{N}_s+1,p{N}_s+{N}_s\right)$, ${\widehat{k}}_{s,p}\in {Z}^{+}$, record ${\widehat{k}}_{s,p}$. If ${\widehat{k}}_{s,p}=p{N}_s+1$ or ${\widehat{k}}_{s,p}=p{N}_s+{N}_s$, reselect the data from *pN*_*s*_ + 1 + *c* to *pN*_*s*_ + *N*_*s*_ + *c* and find the corresponding peak position ${\widehat{k}}_{s,p}^{(ag)}$ according to the reselected data and (29). Decide whether the edge of the (*p* + 1)th data segment covers the phase change point and record ${\widehat{k}}_{s,p}^{(ag)}$. If the edge covers the phase change point, record ${\widehat{k}}_{s,p}=p{N}_s+{N}_s$ or ${\widehat{k}}_{s,p+1}=\left(p+1\right){N}_s+1$; otherwise, set ${\widehat{k}}_{s,p}$ as a null value. Then, *p* = *p* + 1. If *p* < Num − 1, continue this step and process the next data segment;
5. Finish the phase change point estimation of the Num data segments and obtain ${\widehat{k}}_{s,p}$ and ${\widehat{k}}_{s,p}^{(ag)}$, *p* = 0, …, Num − 1, where ${\widehat{k}}_{s,p}$ is the phase change point estimation in the (*p* + 1)th data segment and ${\widehat{k}}_{s,p}^{(ag)}$ is the recorded position of the phase change point estimation result using the reselected data of the (*p* + 1)th segment. In order to make full use of the estimation results, use ${\widehat{k}}_{s,p}$ and ${\widehat{k}}_{s,p}^{(ag)}$, *p* = 1, …, Num − 1, to modify the false phase change points which are caused by the noise. For the recorded ${\widehat{k}}_{s,p}^{(ag)}$, if ${\widehat{k}}_{s,p+1}\in \left(p{N}_s+1,p{N}_s+1+c\right)$ and ${N}_s-c+{\widehat{k}}_{s,p+1}\ne {\widehat{k}}_{s,p}^{(ag)}$, ${\widehat{k}}_{s,p+1}$ is regarded as a false position and mark it as a null value. Finally, we obtain all the phase change points in the LFM/BPSK signal;
6. Omit the estimation result of the first data segment and the number of the rest estimated phase change points is Num − 1. Find the nearest two ${\widehat{k}}_{s,p}$ which are not null and mark them as ${\widehat{k}}_{s,{p}_1}$ and ${\widehat{k}}_{s,{p}_2}$, where *p*_2_ > *p*_1_. Thus the code length of the hybrid modulated signal is ${L}_B=\left({p}_2-{p}_1\right){N}_s+{\widehat{k}}_{s,{p}_2}-{\widehat{k}}_{s,{p}_1}$.

## Simulation results

In this section, numerical simulations are conducted to demonstrate the merits of the proposed scheme. SFM signal is adopted as the LOS modulation for the NYFR, and the LOS modulation can be expressed as *m*(*t*) = 2*πf*_*s*_*t* + *m*_*f*_ sin(2*πf*_sin_*t*) + *φ*_LOS_, where the LOS carrier frequency *f*_*s*_ is 1 GHz, the LOS modulation coefficient *m*_*f*_ is 4, the LOS modulation frequency *f*_sin_ is 10 MHz, the LOS initial phase *φ*_LOS_ is 0, and the number of the monitored Nyquist zones is 10.

The hybrid modulated LFM/BPSK signal is $s(t)=A{e}^{j2\pi {f}_ct+j\pi {\mu}_0{t}^2+j\phi (t)+j{\varphi}_0}$, where the carrier frequency *f*_*c*_ is 4.1 GHz, the chirp rate *μ*_0_ is 50 MHz/μs, the BPSK modulation *ϕ*(*t*) is [-1 -1 1-1 -1 1 1-1 1 1], the signal amplitude *A* is 1, the signal initial phase *φ*_0_ is 0, the signal length is 1 μs, and the ADC sampling rate *f*_ADC_ is 2 GHz.

### Chirp rate estimation simulation

**Fig. 5:**
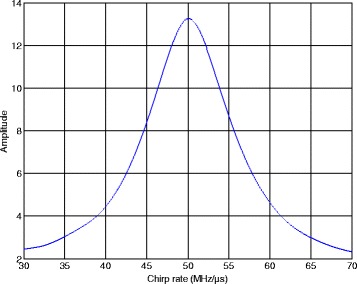
CSVR spectrum of NYFR output signal

The CSVR spectrum of the NYFR output signal based on Section 3.1 is given. Here, we set the signal-to-noise ratio (SNR) as 7 dB and the scanning chirp rate resolution is 0.01 MHz/μs. Considering the LOS parameters, we have *N*_*c*_ = *f*_ADC_/*f*_sin_ = 200 and *M*_*c*_ = *N*/*N*_*c*_ = 10. Hence, the signal can be modeled as a 200 × 10 matrix and the CSVR spectrum is shown in Fig. 5.

From Fig. 5, when the scanning chirp rate is equal to 50 MHz/μs, the CSVR spectrum meets its maximum, which agrees the analysis of (10). In addition, when the scanning chirp rate is far from the signal chirp rate, the non-periodic LFM component will affect the singular values. Hence, the non-principle singular values will increase and ${\overline{\lambda}}_{\mathrm{res}}$ in (11) will rise up. Moreover, according to the energy conservation theory, the principle singular value *λ*_1_ will decrease. Thus, the amplitude of CSVR spectrum will drop with the increasing distance between the scanning chirp rate and the signal chirp rate.

**Fig. 6:**
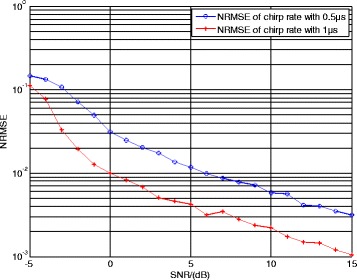
NRMSE of chirp rate estimation with different signal lengths

Figure 6 illustrates the normalized root mean square error (NRMSE) of chirp rate estimation using CSVR spectrum with different signal lengths. The signal lengths are 1 and 0.5 μs, respectively, and other parameters remain unchanged. The number of Monte Carlo experiments is 200.

In Fig. 6, the NRMSE of chirp rate estimation using 1-μs signal length is less than 10^− 2^ when the SNR is greater than 0 dB, which shows a better performance compared with the signal whose length is 0.5 μs. The reason is the bandwidth of LFM component with 1-μs length is wider and its resolution capability is better. This simulation result proves the discussion in Section 3.1.

### NZ index estimation simulation

**Fig. 7:**
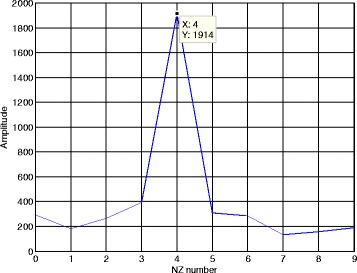
Matching component function

The NZ index estimation result based on the matching component function in Section 3.2 is given in Fig. 7. The SNR is still set as 7 dB. Because the number of Nyquist zones has been set as 10, the argument in *P*_NZ_(*k*) can be set as *k* = 0, 1, ⋯, 9. According to the known *f*_sin_ = 10MHz and *f*_ADC_ = 2GHz, the shift length *τ* can be set as 100 points in (13).

Considering the simulation parameters, the real NZ index should be *k*_NZ_ = round(4.1GHz/1GHz) = 4. From Fig. 7, it is shown when *k* = 4, *P*_NZ_(*k*) achieves its maximum. Apparently, the proposed method can obtain the correct NZ index.

Let us focus on the amplitude value of the peak in Fig. 7. Because the selected signal length in our simulation is *M*_*c*_*N*_*c*_ = 2000, the shift length is *τ* = 100 and the signal amplitude is *A* = 1, the theoretical value of *P*_NZ_(*k*) can be computed as *P*_NZ_(*k*_NZ_) = *A*^4^(*N*_*c*_*M*_*c*_ − *τ*) = 1900 when *k* = *k*_NZ_ = 4 from (17). Meanwhile, the peak value of the simulation result in Fig. 7 is 1914 and we have 1914 ≈ *P*_NZ_(*k*_NZ_). Hence, this simulation proves the correctness of (17). In addition, comparing frequency domain peak search for each channel in [8], the proposed method only needs one-dimensional search for the matching component function and the computational complexity of our method is small. Once the chirp rate and the NZ index are estimated, we can estimate the carrier frequency according to Section 3.2.

**Fig. 8:**
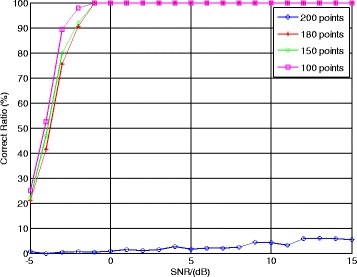
Correct ratio of NZ index using different shift lengths

Besides, in order to examine the shift length selection criterion in Section 3.2, we set different shift length values to show how the shift length affects the correct ratio of NZ index. The values of shift length *τ* are set as 100, 150, 180, and 200 points, respectively. Other parameters remain unchanged. Figure 8 gives the correct ratio of NZ index using a different shift length.

From Fig. 8, the estimation correct ratio of NZ index has the best performance when the shift length *τ* = 100, which implies the distance between *τ* and *f*_ADC_/*f*_sin_ = 200 is the largest. When the distance between *τ* and *f*_ADC_/*f*_sin_ is smaller, the correct ratio of NZ index will decrease. Particularly, when *τ* = *f*_ADC_/*f*_sin_ = 200, the Bessel function in (16) will become *J*_*m*_(⋅)≡0 and the matching component function will lose its capability. This simulation proves our discussion about the shift length selection criterion in Section 3.2.

**Fig. 9:**
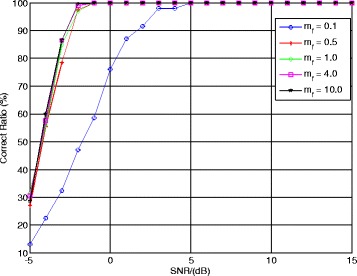
Correct ratio of NZ index using different modulation coefficients

Furthermore, to show the effect of modulation coefficient *m*_*f*_ to the matching component function, different modulation coefficients are used to estimate the NZ index. The values of modulation coefficient *m*_*f*_ are set as 0.1, 0.5, 1, 4, and 10, respectively. Other parameters remain unchanged. Figure 9 presents the correct ratio of NZ index using different modulation coefficient.

From Fig. 9, the correct ratio of NZ index with *m*_*f*_ = 0.1 is less than 90 % when the SNR <3 dB. Meanwhile, the results with other modulation coefficients have better performances and their correct ratios are greater than 90 % when the SNR >−3 dB. The reason of this phenomenon is that the value of the Bessel function in (16) will approach to 1 when |*m*_*f*_| → 0 and the relationship in (18) cannot be guaranteed. When |*m*_*f*_| > 0, the relationship in (18) can be guaranteed, which implies the NZ index estimation performances (except *m*_*f*_ = 0.1) are better and tend to be the same. This simulation proves the discussion about the modulation coefficient selection criterion in Section 3.2.

### Phase change point and code length estimation simulation

**Fig. 10:**
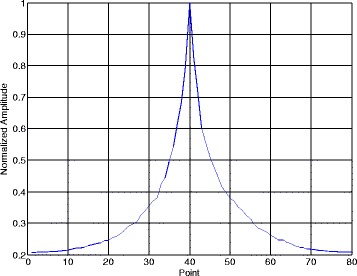
Data segment contains one phase change point

**Fig. 11:**
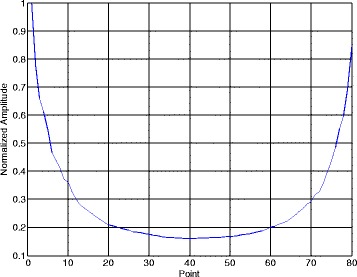
Data segment has no phase change point

Figures 10 and 11 illustrate the normalized phase search pseudo-spectra with phase change point and without phase change point in one segment. The SNR is still 7 dB and the segment length in Section 3.3 is *N*_*s*_ = 80. Figure 10 represents the eighth segment and Fig. 11 is the ninth segment. According to the simulation parameters, the code length of BPSK is 200 points.

**Fig. 12:**
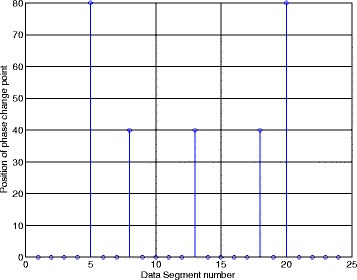
Phase change point in each data segment

From Fig. 10, when ${\widehat{k}}_s=40$, the phase search pseudo-spectrum Phase(*k*_*s*_) reaches its maximum and this peak corresponds to the phase change point of the third and the fourth codes in [-1 -1 1-1 -1 1 1-1 1 1], which means the position of the phase change point is (*p* − 1) × *N*_*s*_ + *k*_*s*_ = (8 − 1) × 80 + 40 = 600. From Fig. 11, Phase(*k*_*s*_) shows a peak at the first point when the data segment has no phase change point. The data in the ninth segment correspond to the signal points from 640 to 720, and obviously, there is no phase change point in such data segment. After we present the phase search pseudo-spectrum in one segment, Fig. 12 gives the estimated phase change point position ${\widehat{k}}_{s,p}$ of the NYFR output signal in each data segment. The number of the data segments in Fig. 12 is Num − 1 = floor(2000/80) − 1 = 24.

The horizontal axis in Fig. 12 indicates the data segment number and the vertical axis represents the position of each phase change point. When the value of vertical axis is 0, it means there is no phase change point in that segment. In Fig. 12, five phase change points have been estimated and the position of each phase change point can be calculated based on the segment length and the vertical axis values. In addition, from the 5th and 20th segments, we can see the edges of these data segments cover the phase change points. However, our method can still estimate these covered phase change points.

### Parameter estimation performance

At last, let us consider the performance of the proposed method. Although there is no public report for the parameter estimation algorithm of the LFM/BPSK signal intercepted by the NYFR, we still can employ the algorithm using multi-channel structure in [8] and the method in [21] as the comparisons. The shift length *τ* is 100 points and the LOS modulation coefficient *m*_*f*_ is 4. The correct ratio of NZ index, the NRMSEs of chirp rate, and carrier frequency and the correct ratio of code length are given, respectively. The SNR is set from −5 to 15 dB, and 500 Monte Carlo trials are used for each SNR value.

**Fig. 13:**
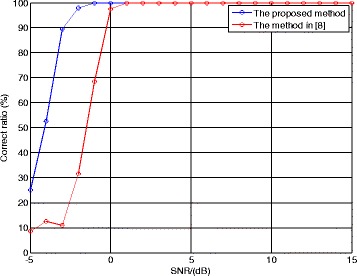
Correct ratio of NZ index

Figure 13 compares the NZ index correct ratio of the proposed method with that of the method in [8]. The proposed method performs better than the method in [8] when SNR <0 dB, because the method in [8] estimates the NZ index using the amplitude value in frequency domain which is sensitive to the noise.

**Fig. 14:**
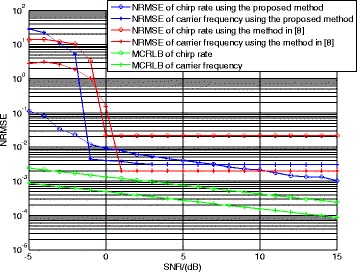
NRMSEs of chirp rate and carrier frequency estimation

Figure 14 illustrates the NRMSEs of chirp rate and carrier frequency estimation results using the proposed method, the method in [8], and the modified Cramer Rao lower bound (MCRLB) [22], respectively. As indicated in the result, when SNR >−2 dB, the chirp rate estimation of our method can achieve NRMSE <0.01. However, the chirp rate estimation method in [8] yields large estimation errors when SNR <0 dB due to the NZ index estimation transfer error. In detail, when SNR <0 dB in Fig. 13, the correct ratio of NZ index of [8] is smaller than 90 % and the NZ index transfer error will lead to a poor chirp rate estimation performance, which can be seen from Fig. 14. In contrast, the proposed method estimates chirp rate directly and its performance will not be affected by the NZ estimation result. In addition, because the CSVR spectrum is a super resolution method, the proposed method is closer to the MCRLB.

Figure 14 also reveals that the proposed method performs better than the method in [8] in estimating the carrier frequency when 2 dB > SNR > −2 dB, because the proposed method has a better NZ index estimation performance. When the SNR is greater than 2 dB, the performances of both methods are almost the same due to the same carrier frequency estimation process (i.e., Fourier transform).

**Fig. 15:**
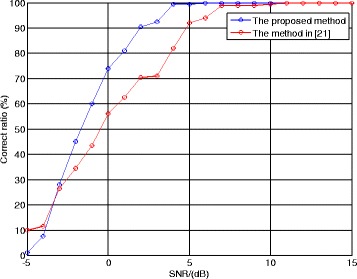
Correct ratio of code length

Figure 15 presents the correct ratio of code length estimation using the proposed method and the method in [21]. Here, the correct ratio of code length means when the code length estimation result strictly equals (1 × 10^− 6^/10) × *f*_ADC_ = 200 points, we regard that the estimation result is correct. From Fig. 15, the proposed method outperforms the method in [21] and the correct ratio of the proposed method is greater than 90 % when SNR >2 dB, because the peak width of phase search pseudo-spectrum is narrower.

In summary, for the LFM/BPSK hybrid modulated signal intercepted by the NYFR, the proposed method can obtain accurate estimation performances for the chirp rate, the carrier frequency, the NZ index, the code length, and the phase change points when SNR is greater than 2 dB.

## Conclusions

On the basis of the NYFR prior information and signal self-characteristic, the parameter estimation algorithm of LFM/BPSK hybrid modulated signal intercepted by the NYFR has been proposed. We make full use of the LOS prior information to model the NYFR output signal and propose the CSVR spectrum to estimate the chirp rate directly. Then, according to the self-characteristic of the SFM modulation, the matching component function has been designed to estimate the NZ index and the carrier frequency. Finally, the matching code and subspace orthogonal property have been employed to obtain the position of each phase change point and the code length. Furthermore, we also analyze the parameter selection criteria and the computational complexity for each step. Comparing the existing NYFR output signal parameter estimation algorithm, the proposed algorithm avoids constructing multi-channel architecture and estimating the NZ index firstly. Meanwhile, the proposed scheme can achieve a higher accuracy compared with the existing parameter estimation methods. The simulation results show the proposed scheme demonstrates a good performance and prove our analyses. Besides, the estimation methods in Sections 3.1 and 3.2 can be used to estimate the parameters of LFM signal intercepted by the NYFR in one NZ as well.

## Abbreviations

ADC: Analog to digital converter
BPSK: Binary phase shift keying
CSVR: Chirp singular value decomposition ratio
FA: Frequency agile
LFM: Linear frequency modulation
LOS: Local oscillator
LPI: Low probability interception
MCRLB: Modified Cramer Rao lower bound
NRMSE: Normalized root mean square error
NYFR: Nyquist folding receiver
NZ: Nyquist zone
RF: Radio frequency
SFM: Sinusoidal frequency modulation
SNYFR: Synchronous NYFR
SVD: Singular value decomposition
ZAM: Zhao, Atlas, and Marks
ZCR: Zero crossing rising
